# One-Year Surveillance of SARS-CoV-2 Exposure in Stray Cats and Kennel Dogs from Northeastern Italy

**DOI:** 10.3390/microorganisms11010110

**Published:** 2022-12-31

**Authors:** Laura Bellinati, Mery Campalto, Elisa Mazzotta, Letizia Ceglie, Lara Cavicchio, Monica Mion, Laura Lucchese, Angela Salomoni, Alessio Bortolami, Erika Quaranta, Jacopo Magarotto, Mosè Favarato, Laura Squarzon, Alda Natale

**Affiliations:** 1 Istituto Zooprofilattico Sperimentale delle Venezie, 35020 Legnaro, Italy; 2 ULSS 3 Serenissima, 30174 Venice, Italy; 3 UOSD Genetica e Citogenetica e Diagnostica Molecolare-Azienda ULSS 3 Serenissima, 30174 Venice, Italy

**Keywords:** SARS-CoV-2, stray cats and kennel dogs, exposure COVID-19

## Abstract

Dogs and cats are susceptible to severe acute respiratory syndrome coronavirus 2 (SARS-CoV-2). During the pandemic, several studies have been performed on owned cats and dogs, whereas limited data are available on the exposure to stray animals. The objective of this study was to investigate the exposure to SARS-CoV-2 of feral cats and kennel dogs in northeastern Italy, through serological and molecular methods. From May 2021 to September 2022, public health veterinary services collected serum, oropharyngeal, and rectal swab samples from 257 free-roaming dogs newly introduced to shelters, and from 389 feral cats examined during the routinely trap–neutered–return programs. The swabs were analyzed for viral RNA through a real-time reverse transcriptase PCR (rRT-PCR), and sera were tested for the presence of the specific antibody against SARS-CoV-2 (enzyme-linked immunosorbent assay). Serology was positive in nine dogs (9/257) and three cats (3/389), while two asymptomatic cats tested positive to rRT-PCR. One cat turned out to be positive both for serology and molecular analysis. In addition, this study described the case of a possible human-to-animal SARS-CoV-2 transmission in a cat that travelled in close contact to a COVID-19-positive refugee from Ukraine. This study shows that SARS-CoV-2 can infect, in natural conditions, stray cats and kennel dogs in northeastern Italy, although with a low prevalence.

## 1. Introduction

*Coronaviridae* is a family of enveloped single-stranded positive-sense RNA viruses that cause respiratory and intestinal infections in animals and humans [1]. In December 2019, a novel coronavirus of the genus *Betacoronavirus*, subgenus *sarbecovirus*, named severe acute respiratory syndrome coronavirus 2 (SARS-CoV-2) emerged in the city of Wuhan, China, and caused an outbreak of unusual viral pneumonia [2,3]. With a high human-to-human transmissibility, this novel coronavirus disease, also known as coronavirus disease 2019 (COVID-19), has rapidly spread worldwide causing a pandemic. SARS-CoV-2 is the third most fatal respiratory infection caused by coronaviruses that has emerged in humans, after severe acute respiratory syndrome coronavirus (SARS-CoV) in 2002 [4] and Middle East respiratory syndrome coronavirus (MERS-CoV) in 2012 [5]. Both SARS-CoV and MERS-CoV originated in bats but had intermediate hosts, respectively, palm civets (*Paradoxurus hermaphroditus*) [6] and dromedary camels (*Camelus dromedarius*) [7], and only subsequently did they infect humans. The same process is believed to have occurred for SARS-CoV-2 [8,9]; indeed, the most related coronaviruses were found in bats but, currently, the reservoir hosts of the virus have not been clearly proven. In truth, it is still unknown whether there was a SARS-CoV-2 intermediate host and which animal may have been able to establish itself as such [10,11]. Furthermore, previous studies have shown that genetic recombination may have originated some sarbecoviruses; it cannot be excluded that viral RNA recombination of several related coronaviruses played an important role in the evolution of SARS-CoV-2 [12].

Coronaviruses are characterized by a high mutation rate [13] and frequent homologous recombination [14], which have contributed to a great genetic diversity and allowed the infection of numerous animal species [15,16]. The rapid spread of SARS-CoV-2 and its mortality rate have raised concern about the potential capacity of the virus to adapt to other species and become more transmissible and virulent after mutations occurring in the infected animals [17]. Since the early stage of the pandemic, the computational analysis in silico of the virus’s natural receptor angiotensin converting enzyme 2 (ACE 2) indicated that SARS-CoV-2 is able to infect different animal species [18]. This was subsequently confirmed with in vivo studies [19] and by the detection of natural infections linked to human exposure in various animal species, including felines, canines, mustelids, and primates [20,21]. Among these species, dogs and cats are the most common companion animals worldwide and several studies have indicated that both are naturally and experimentally susceptible hosts to SARS-CoV-2 [22,23]. Human-to-animal transmission followed by animal-to-animal circulation of the virus associated with genetic evolution amongst several animal species has been documented for several wild or farmed species. Transmission of the virus from infected owners to their cats and dogs has also been frequently reported [24,25,26,27,28,29,30].

Stray cats and dogs and colony cats have a great ecological impact due to their interaction with other urban and periurban animals [31,32]. Their epidemiological role in the COVID-19 pandemic in Europe and other countries has been analyzed in different studies and a broad range of prevalence was found [33,34,35,36,37,38]. SARS-CoV-2 in domestic cats and dogs mostly showed the absence of prominent clinical signs [28,39] but stray animals do not receive the same veterinary and preventive care as domestic pets, possibly resulting in serious health issues if infected with concomitant diseases [36,40]. In addition, recently, a suspected case of SARS-CoV-2 cat-to-human transmission has been reported [41]. As the epidemiological role of cats and dogs in the COVID-19 pandemic is still not fully understood, the monitoring and evaluation of SARS-CoV-2 infection in stray animals could represent a crucial point to achieve knowledge on the degree of SARS-CoV-2 diffusion in susceptible animals with limited human contact. In a One Health perspective, this approach could lead to a better understanding of the possible spillover events related to close contacts of exposed people (feline colony and shelter caregivers and the veterinary service personnel) with free-roaming dogs and cats.

In addition, since the start of the COVID-19 pandemic, numerous SARS-CoV-2 variants have emerged, whose changes in the viral genome have occurred in different subjects (humans/animals) and in the transmission of the virus between susceptible hosts [42,43]. The determination of the susceptibility of various animal species to infection with SARS-CoV-2 and the risks associated with the circulation of SARS-CoV-2 in animals [44] remains crucial to inform the appropriate human and to correct the veterinary public health responses to this pandemic.

The aim of the present study was to assess the viral circulation and serological positivity prevalence of SARS-CoV-2, both in free-roaming dogs newly introduced to shelters and in stray cats captured for the “trap–neutered–return” (TNR) program in northeastern Italy, to better characterize the relevance of stray animals in the pandemic and determine the possible risk of spillover events.

In addition, we report a case of identification and characterization of SARS-CoV-2 in a cat that travelled from Ukraine to Italy in close contact with a COVID-19-positive refugee.

## 2. Materials and Methods

### 2.1. Sample Population

The study was carried out in seven provinces of the Veneto and Trentino-Alto Adige regions. Between May 2021 and September 2022, serum, oropharyngeal (OP) and rectal (R) swab samples were collected from a total of 257 free-roaming dogs and subjects newly introduced to shelters, as well as from 389 stray cats. Sampling was performed by veterinarians for diagnostic, therapeutic or prophylactic purposes. Animal care and procedures were carried out in compliance with the Guide for the Care and Use of Laboratory Animals and Directive 2010/63/EU for animal experiments (National law: D.L. 26/2014), and the study received ethical committee approval: CE_IZSVE 8/2020.

The samples were refrigerated (+4 °C) and shipped to the Istituto Zooprofilattico Sperimentale delle Venezie (IZSVe) for molecular and serological analyses. The samples were then stored at −80 °C for possible further investigations.

Epidemiological and anamnestic data were collected for the enrolled animals, including sexual status (neutered/intact sex), age, breed, clinical symptoms, medical condition, and province of origin.

### 2.2. Molecular Investigation

The collected OP and R swabs were cut in sterile microtubes filled with 1 mL of phosphate-buffered saline 1X (PBS), mixed by vortexing, and stored at −80 °C. RNA was extracted by the KingFisher™ Flex Purification System instrument (Life Technologies, Carlsbad, CA, USA) using the ID Gene^®^ Mag Universal Extraction Kit (ID.vet, Grabels, France), in accordance with the manufacturer’s instructions, adding a pretreatment with 20 µL of Proteinasi K (QIAGEN, Hilden, Germany) and 250 µL of the Lysis provided by the kit, for 10 min at 70 °C before the extraction. Every RNA extraction included a negative control (water).

The eluted RNAs were subjected to the specific SARS-CoV-2 rRT-PCR method described by Corman et al. [45]. In previous studies, cross-reactivity of the test utilized was assessed for common human coronaviruses (HCoV-229E, HCoV-NL63, and HCoV-OC43), MERS-CoV, SARS-CoV, feline coronavirus (FCoV TN406HP), feline infectious peritonitis virus (FIPV), canine coronavirus (CCV-378), and bovine coronavirus (BCV), reporting good performance and no cross-reactivity [45,46]. In addition, R swabs from SARS-CoV-2-positive cats were further tested for FCoV and all resulted negative.

The screening of all samples was performed in a pool of eluted RNA of 10 animals, only targeting the envelope protein (E) gene fragment on a CFX 96 Deep Well Real time PCR system (Bio-Rad Laboratories Inc., Hercules, CA, USA). Results were analyzed with the Bio-Rad CFX Maestro 1.1 software (Bio-Rad Laboratories Inc., Hercules, CA, USA) and, following an in-house validation with quantified plasmids, the Ct values cut-off of 40.0 was adopted. A universal heterologous control RNA, Intype IC-RNA (Indical Bioscience GmbH, Leipzig, Germany) was added to each sample in the extraction step with a ratio of 1:10 of the total elution volume and amplified by using the primers and probe as per Hoffman et al. [47] in order to check the efficiency of the RNA extraction and validate each negative result.

Whenever a positive sample was found, all the single RNA of the pool were analyzed for the E gene fragment, subsequently, the positive samples were analyzed also for the nucleoprotein (N) and RNA-dependent RNA polymerase (RdRp) encoding gene fragments.

### 2.3. Serological Investigation

The specific serological response against SARS-CoV-2 was investigated in the sera samples from cats and dogs with an enzyme-linked immunosorbent assay (ELISA) commercial kit. In the case report of a suspected SARS-CoV-2-positive cat from Ukraine, the plaque reduction neutralization test (PRNT) was also performed to better evaluate the serological response.

#### 2.3.1. Enzyme-Linked Immunosorbent Assay (ELISA)

*ID Screen^®^-SARS-CoV-2 Double Antigen Multi-species* (ID.vet, Grabels, France) detects antibodies against the nucleocapsid (N) protein (ELISA KIT). The wells are coated with purified N proteins that form a complex in the presence of antibodies against the virus. Following the manufacturer’s instructions, samples were diluted at 1:20 and incubated at 37 °C, before adding a purified N protein antigen horseradish peroxidase (HRP)-conjugate, which can bound the free Fab of the antibodies and led to the colorimetric reaction. When the reaction stop, the O.D. values read at 450 nm were used to calculate the S/P% as described in the manufacturer’s instructions. If the validation criteria are respected, the sample is considered negative with SP% ≤ 50%, positive with SP% ≥ 60% and doubtful with a value in-between. The test is validated for multi-species use, as the double-antigen method is species-independent.

#### 2.3.2. Plaque Reduction Neutralization Test (PRNT)

PRNT assays were performed in a Biosafety Level 3 (BSL3) laboratory using a SARS-CoV-2 isolate, as previously described [48]. In brief, serum samples were heat-inactivated (56 °C for 30 min) and 2-fold diluted in Dulbecco-modified Eagle medium (DMEM). Serum dilutions were mixed with an equal volume (1:1) of a virus solution containing approximately 25 focus-forming units (FFUs) of SARS-CoV-2 and incubated for 1 h at 37 °C in a 5% CO_2_ incubator. Fifty microliters of the virus–serum mixtures were added to the confluent monolayers of Vero E6 cells, in 96-wells plates and incubated for 1 h at 37 °C, in a 5% CO_2_ incubator to allow for the infection of the cells. A total of 100 L of an overlay solution made of minimum essential medium (MEM) with 2% fetal bovine serum (FBS, Sigma, Saint Louis, MO, USA), penicillin (100 U/mL, Sigma, Saint Louis, MO, USA), streptomycin (100 U/mL, Sigma, Saint Louis, MO, USA), and 0.8% carboxy methyl cellulose (CMC, Sigma, Saint Louis, MO, USA) were then added to each well after inoculum removal. After 26 h of incubation, the overlay was removed, and the cells were fixed with a 4% paraformaldehyde (PFA) solution. The visualization of the plaques was obtained with an immunocytochemical staining method using an anti-Nucleoprotein monoclonal antibody (1:10,000; Sino Biological Inc., Beijing, China) for 1 h, followed by 1 h incubation with peroxidase-labelled goat anti-mouse antibodies (1:1000; Dako, Glostrup, Denmark) and a 7 min incubation with the True Blue (KPL, Gaithersburg, MD, USA) peroxidase substrate. FFUs were counted after the acquisition of the pictures BioSpot™ (CTL Europe GmbH, Bonn, Germany). Tests were run in triplicate.

The neutralization titre was defined as the reciprocal of the highest dilution resulting in a reduction in the control plaque count > 50% (PRNT_50_).

### 2.4. Case Report of A Cat of A Positive Owner from Ukraine

In March 2022, a 5-year-old intact male European shorthair cat was admitted to a shelter in the province of Venice because its owner, a COVID-19-positive refugee from Ukraine, was receiving healthcare in quarantine facilities, as required by the regulations in force at the time. The physical examination of the cat was unremarkable, the referring veterinarians reported as the only clinical issue, the presence of gastrointestinal tapeworm (*Dipylidium caninum*). OP and R swabs, as well as serum were collected for the molecular and serological analysis at three different time points:

- T0: Cat’s first admission to the shelter.
- T1: 7 days after T0.
- T2: 30 days after T0.

Since the cat came from a COVID-19-positive household, additional shipment (UN3373) and manipulation measures were adopted: the collected swabs were put in Primestore ^®^ MTM (Longhorn Vaccines & Diagnostics LLC, Bethesda, MD, USA) in the BSL3 facility.

At T0, the swabs were processed for molecular analysis of SARS-CoV-2 and resulted positive. The cat was kept in a quarantine area of the shelter to be checked for any possible clinical symptoms, and to avoid the spread of the virus. OP and R swabs and the serum sample were collected after one week (T1) for molecular and serological (ELISA and PRNT) analyses, as described above. The serum sample was re-tested after one month (T2) through the same protocol.

### 2.5. Sequencing Analysis

Complete genome sequencing was performed on the RNAs extracted from the positive OP and R swabs sampled in the cat described in the case report. Sequences were obtained using an Illumina MiSeq platform (Illumina, San Diego, CA, USA) and an in-house protocol for target amplification. After trimming and filtering for quality, reads were aligned against the reference genome (GenBank: NC_045512.2) using BWA-mem [49,50]. The sequences were deposited in GISAID under accession numbers EPI_ISL_15909005: hCoV-19/cat/Italy/VEN-IZSVe-22RS577-1_VE/2022 (OP) and EPI_ISL_15909031: hCoV-19/cat/Italy/VEN-IZSVe-22RS577-10_VE/2022 (R). The virus lineage was assigned according to the PANGOLIN application (https://pangolin.cog-uk.io/, Rambaut et al., 2020) (accessed on 4 November 2022) [51,52].

### 2.6. Statistical Analysis

Laboratory results, epidemiological, and anamnestic data per individual cat or dog were analyzed using the statistical software R (version 4.2.1, https://www.R-project.org/, accessed on 25 October 2022). The animals were grouped according to their age (<1 y/o, from 1 to 5 and >5 y/o, from 5 to 10 y/o, and >10 y/o,) and their breeds (cross-breed, hunting dogs (i.e., Épagneul Breton, Cocker Spaniel, Hound, Gordon Setter, English Setter, Jag Terrier, Poodle, Deutsch Drahthaar, Jack Russell Terrier, and Dachshund), herding dogs (i.e., German Shepherd, Lagorai Shepherd, and Czechoslovakian Wolfdog) and molossoid dogs (i.e., American Staffordshire Terrier, Corso, Dogo Argentino, Pinscher, Pit Bull, and Rottweiler Mastiff).

The demographic and clinical data of the population, the prevalence of positive animals with a 95% confidence interval (95% CI) and the statistical relevance evaluated with Pearson’s Χ^2^ test, were performed.

The open source software QGIS 3.16 (http://www.qgis.org, accessed on 25 October 2022) was used to construct a map to compare free-ranging and shelter animals’ SARS-CoV-2-positive cases with COVID-19 human cases. The data on the human cases were collected from the Italian Ministry of Health (https://www.salute.gov.it, accessed on 25 October 2022).

## 3. Results

### 3.1. SARS-CoV-2 Serological Investigation and Viral Detection in Free-Roaming/Shelter Dogs and Free-Ranging/Colony Cats

Samples were collected from a total of 257 dogs and 389 cats in the period between May 2021 and September 2022 in seven provinces of northeastern Italy (Bozen and Trento in the Trentino-Alto Adige region and Padua, Rovigo, Venice, Vicenza, and Verona in the Veneto region). Serological analysis in dogs reported positivity in eight animals (S/P% between 101% and 356%, mean of 160%). One sample reported borderline value (S/P% = 53%). The overall seroprevalence reported in the dogs’ population during the study period was 3.5% (95% CI 1.6–6.5).

Three hundred and seventy-nine cats’ sera were negative, and three cats resulted positive, reporting a seroprevalence of 0.8% (95% CI 0.2–2.2).

The correlation of the anamnestic and epidemiological data and serological positivity did not report any statistically significance in both groups (*p* > 0.05). The difference in the seroprevalence between cats and dogs is statistically significant with *p* < 0.05 (Pearson’s Χ^2^ test: Χ^2^ = 6.675, df = 1, *p*-value = 0.0098). The epidemiological information, serology and the statistical results are summarized in Table 1.

The characteristics of the seropositive dogs and the serological ELISA value (S/P%), are detailed in Table 2. All dogs were neutered, the majority was male (66.7%), 56% were puppies (<1 y/o), 22% were young dogs < 5 y/o, one dog (11%) was an adult (7 y/o) and one dog (11%) was senior (>10 y/o). Most of the dogs were crossbreeds (88.9%), one dog was a Hound and one was a Pit Bull. All the dogs were asymptomatic on the date of samples’ collection. Padua, Venice, and Vicenza are the provinces that reported positive animals.

The 257 dogs resulted negative for molecular analysis, both in OP and R swabs.

Molecular analysis on 389 feral cats found two positive OP swabs (0.5%, 95% CI 0.1–1.8): rRT-PCR analysis detected positivity on gene E and N in one cat, while another one resulted positive to genes E, N and RdRp. One cat reported positivity for SARS-CoV-2 molecular assay and serum antibodies simultaneously.

The description of the positive cats, the Ct value of rRT-PCR and the SP% value of the ELISA analysis are reported in Table 3. Most of the positive subjects were male cats (75%), 50% were very young animals (1 y/o), one cat was 2.5 y/o and one cat was 8.5 y/o. All the positive cats were not neutered/spayed. Three positive cats (75%) came from the province of Venice, and the two resulted positive to rRT-PCR were collected in the same province and one week apart.

#### Ricerca Corrente 12/19 (Current Research—Italian Ministry of Health—n. 12/19) Serology and Molecular Results: Comparison with COVID-19 Human Cases

Out of the 646 stray animals collected, 389 stray cats and 257 kennel dogs, 12 samples (1.9%, 95%CI 1–3.2) were positive for anti-SARS-CoV-2 antibodies, and two samples (0.3%, 95%CI 0–0.11) resulted positive for SARS-CoV-2 RNA. 

**Figure 1:**
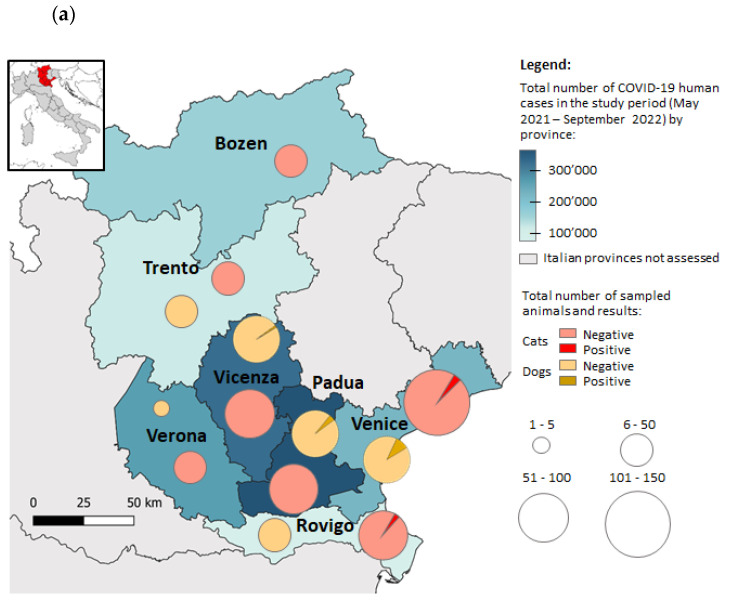

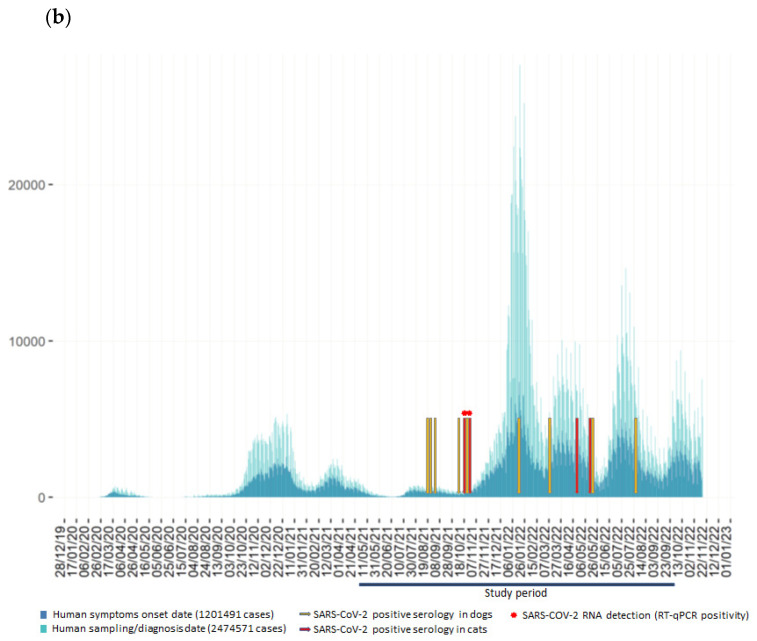
(**a**) Outline of the Italian provinces participating in the study, the color scale identified the total number of human cases reported during the study period from May 2021 to September 2022 (Italian Ministry of Health: https://www.salute.gov.it, accessed on 25 October 2022). Distributions and result of the sampled animals by province are shown. (**b**) Details of the serological and molecular positive sample in dogs and cats, compared to the curve of daily new cases reported in the Veneto region during the study period.

The total number of COVID-19 human cases reported during the study period (May 2021–September 2022) in the investigated Veneto and Trentino Aldo-Adige provinces, the number of sampled animals and the positivity to the molecular and/or serological analyses are reported in Figure 1a.

Details of the serological and molecular positive cases identified in dogs and cats, compared to the number of human cases in the Veneto region during the study period are pictured in Figure 1b (https://www.salute.gov.it/imgs/C_17_monitoraggi_147_20_fileRegionale.pdf (accessed on 22 November 2022)).

The highest number of positive animal samples, 88.9% dogs and 100% cats, were recorded in the provinces with the highest number of human-positive cases (Padua, Venice and Vicenza).

Most of the seropositive samples reported in the present study, respectively, 44% in dogs and 50% in cats, occurred within a short period of time following the increase in COVID-19 human cases. The two cats resulted positive to the molecular analysis and one seropositive dog was sampled before the 2021 winter season, at the beginning of the highest human COVID-19 wave in the regions under investigation. Interestingly, 44% of the positive serum samples from dogs were collected when the human cases were at their lowest since the beginning of 2021.

### 3.2. Case Report of a SARS-CoV-2 Detection and Characterization in A Cat and COVID-19 Positive Owner

At the first time point (T0), in March 2022, the OP and R swabs resulted positive to rRT-PCR assay targeting the SARS-CoV-2 RNA nucleoprotein gene and envelope protein gene, and both were negative to the RdRp gene. At this time point no serological analysis was performed.

Seven days later (T1), OP/R swabs and sera were collected from the cat. In both swabs no rRT-PCR positivity was detected, and also the ELISA and PRNT test turned out to be negative. One month later (T2), the serum sample tested negative to both the ELISA and PRNT test (Table 4).

The characterization of the complete genome of SARS-CoV-2 from OP and R swabs collected from the cat, and from the nasal swab of its owner (sequence generated at UOSD Genetics and Cytogenetics—Azienda ULSS 3 Serenissima) revealed that the virus identified in all three swabs belonged to the Omicron BA.2.9 *sublineage* (Pangolin v.4.0.5, Scorpio v.0.3.16, pangolin data v.1.3). The BA.2.9 *sublineage* belongs to the BA.2 *lineage*, which was present worldwide and prevalent in Italy at that time.

A 61% and 89% consensus sequence were obtained for the OP sample and R swab of the cat, respectively, while an 89% consensus sequence was obtained for the owner’s nasal swab.

The genome of the cat’s R swab virus and that of the owner’s nasal swab differed by two nucleotide substitutions, one of which resulted in an amino acid mutation in ORF1b (F685Y). The viral genomes analyzed had two amino acid substitutions in ORF1a (K822R and Q1021E), respectively, present in the 0.04% and 0.06% of the BA.2.9 sequences in GISAID (25/11/2022).

## 4. Discussion

Several studies have demonstrated the susceptibility of domestic animals to SARS-CoV-2, both with experimental and natural infection. Natural human-to-pet SARS-CoV-2 transmission seems to be most commonly due to the close contact between pets and COVID-19 owners (COVID-19-positive households) [53,54].

Considering new evidence confirming that pet infection rates could change on a SARS-CoV-2 variant basis [55], which could cause a selection of variants of concern for humans [56], a “One Health” surveillance approach to track virus prevalence in domestic, wild, and stray animals is deemed necessary to determine the impact that animals had, have, and will have in the COVID-19 pandemic, and to assess their influence on transmission to human [57,58,59].

Indeed, epidemiological studies of the seroprevalence of SARS-CoV-2 in stray animals reported a lower prevalence than the one observed in owned animals. Different studies analyzed the seroprevalence of stray cats ranging between 0% and 3.5% [33,34,35,36,37,38,60]; conversely, only a few studies have analyzed serological positivity for SARS-CoV-2 in free-roaming/shelter dogs [38,60]. Recently, Cardillo et al. [60] described a higher seroprevalence in stray dogs (1.28%) rather than in cats (0.42%) in the Campania region (southern Italy), whereas this trend is reversed in owned animals of the same region. Studies of SARS-CoV-2 antibodies in cats in northern Italy reported a seroprevalence between 0% and 1% [34,37].

In the present study, the SARS-CoV-2 molecular detection and serological survey were investigated in 389 free-ranging cats and 257 kennel dogs in a period from May 2021 to September 2022.

The seroprevalence found in cats was 0.8% and for free-roaming dogs it was 3.5%, reporting a statistically significant (*p* < 0.05) difference between the species; however, no correlation with the epidemiological or anamnestic characteristics recorded was identified.

It has been reported that cats seem to be more susceptible to SARS-CoV-2 and that household cats presented higher seroprevalence than domestic dogs [22,61,62]. However, in our study the highest seroprevalence was detected in free-roaming dogs, as reported by Cardillo et al. [60]. This apparent contradiction is probably due to the inclusion criteria of the population, since the dogs had recently been introduced to a shelter and were, therefore, more likely to have come in contact with humans (i.e., their former owner, the veterinary personnel or the shelter caregivers), compared to the stray cats analyzed, which are usually less prone to close contact with people, except for colony caregivers. Additional assumed routes of infection for stray animals are represented by the contact with other infected animals [63] or by exposure to a contaminated environment, such as public surfaces and the sewage system [64,65].

Interestingly, in the current study the serological and molecular positivity of SARS-CoV-2 in animals was detected mainly in the provinces most affected by the pandemic and shortly after an increase in the number of human COVID-19 cases. This supports the assumption that animals are mostly infected by humans (reverse zoonosis), although the circulation of the virus among animals in close contact with each other cannot be excluded [59].

Previous studies reported a higher seroprevalence of SARS-CoV-2 in male unneutered pets, which suggests a behavioral or hormonal aspect to the infection with SARS-CoV-2 [20,53,60]. Although we report a higher number of positive animals in male cats and dogs, our findings revealed no statistical evidence that could confirm a higher seropositive proportion in male pets; the significance could have been biased by the small number of positive animals; thus, gender susceptibility in pets should be further investigated.

Differently from the previous literature [34,37], the present study detected the virus in stray cats by molecular analysis, even though the detected prevalence of SARS-CoV-2 is low (0.5%). It has been described that cats naturally or experimentally infected with SARS-CoV-2 are able to transmit the virus to other cats within two days after the contact, and that the shedding of the virus most likely occurs through the respiratory and gastrointestinal tract [39,66], despite the viral shedding seemingly being for a short period of time [67].

Furthermore, in the current study we present a case report of a SARS-CoV-2-positive cat that travelled in close contact with a COVID-19-positive Ukrainian refugee. SARS-CoV-2 experimentally infected cats show seroconversion about 7–13 days from infection, and they can maintain high neutralizing antibodies titers 42 days after infection [22,39]. Serological positivity has been reported in household cats up to 54 days after diagnosing COVID-19 in the owner [68]. The limited data currently available suggest that the duration of seroconversion following natural infection in cats can produce a detectable antibody titer up to 110 days post-infection [69]. In our study, the cat was positive for SARS-CoV-2 viral detection but did not show any detectable specific antibodies at different time points. Moreover, in our specific case the virus was no longer detectable after one week: this finding suggests a possible short viremia, as reported in naturally infected cats [53,62]. Furthermore, the negativity both in ELISA and PRNT analysis, even after one month from the initial SARS-CoV-2 positivity, indicates a lack of seroconversion. This may be suggestive of mild infections as reported in humans [70] and cats [71], with insufficient stimulation of the cat’s immune system to elicit a detectable humoral immune response [70,72].

Full genome sequence analysis performed on the two swabs collected from the cat and on the nasal swab of its owner identified a SARS-CoV-2 virus belonging to the Omicron BA.2.9 *sublineage*, which is related to the BA.2 lineage circulating with high prevalence in Italy and worldwide at the time of infection. Although amino acid differences were found in the sequences obtained from the samples derived from the cat and its owner, these mutations occur in positions within the viral genome that are not considered relevant for virus fitness and pathogenicity.

Different assumptions have been made on the origin of the Omicron variant, with various studies pointing to spill-back in rodents, followed by inter-species recombination, accumulation of mutations to then finally jump back into humans, all this indicating an inter-species evolutionary trajectory for this variant [73,74]. Despite the fact that Omicron’s roots are far from being conclusively assigned to a specific source, findings from the available scientific literature and from case reports underscore the fact that SARS-CoV-2 is able to bounce back and forth between wildlife, domesticated animals, and the human world (https://vis.csh.ac.at/sars-ani/#infections (accessed on 30 November 2022)). In this context, the importance of SARS-CoV-2 surveillance in companion animals, which may play a role as bridge species between potential wild reservoir candidates and humans, should be not underestimated.

## 5. Conclusions

This study provides serological evidence for a limited SARS-CoV-2 infection in free-roaming dogs and stray cats in northern Italy. The positivity at the molecular analysis observed in cats showed that the virus was able to infect this species, particularly shortly after the increase in COVID-19 human cases. Regardless, the low seroprevalence indicates that free-ranging stray or shelter dogs and feral cats probably did not play a role in the transmission of SARS-CoV-2 during the pandemic. This study contributes to the confirmation of the fact that there is insufficient evidence that stray dogs and cats can represent a source of SARS-CoV-2 infection for people or other pets. Nevertheless, they should be considered important environmental sentinels in the detection of zoonotic agents. Moreover, the monitoring of dogs and cats represents an opportunity to identify potential variations or changes in the characteristics of the pathogens of interest.

## Figures and Tables

**Table 1:** Demographic information and serological results of the enrolled animals. Prevalence and 95%CI (confidential intervals). The statistical relevance and inter-parametric evaluation were performed with Pearson’s Χ^2^ test (*p*-values). Number of missing information are not shown.

	Dogs (257) N (%)	95% CI	Positive	*p*	Cats (389) N (%)	95% CI	Positive	*p*
**Sex**								
Male	144 (57.1%)	51.0–63.2	6	0.94	139 (37.3%)	32.2–41.8	3	
MN	20 (7.9%)	4.6–11.7			36 (9.2%)	6.3–12.1		0.16
Female	77 (30.5%)	28.1–40	3		144 (37.2%)	32.2–41.8		
FN	11 (4.38%)	1.8–6.8			53 (13.6%)	10.2–17.3		
**Age (years)**								
<1	62 (24.1%)	19–29.8	5	0.23	114 (29.3%)	24.8–34.1	1	
1–5	104 (40.5%)	34.4–46.7	2		187 (48.1%)	43–53.2	1	0.16
5–10	43 (16.7%)	12.4–21.9	1		16 (4.1%)	2.4–6.6	1	
>10	25 (9.7%)	6.4–14	1		3 (0.8%)	0.2–2.2		
**Breed**					European shorthair 389 (100%)	99.1–100		n.a.
Crossbreed	213 (82.9%)	77.7–87.3	8	0.85				
Hunting dogs	22 (8.6%)	5.4–12.7						
Herding dogs	5 (1.9%)	0.6–4.5						
Molossoid dogs	17 (6.6%)	3.9–10.4						
**Provinces**								
Bozen	0 (0%)	0–1.4	-		16 (4.1%)	2.4–6.6		
Padua	76 (29.6%)	24.1–35.6	3		67 (17.2%)	13.6–21.4		
Rovigo	21 (8.2%)	5.1–12.2		0.14	58 (14.9%)	11.5–18.8	1	0.78
Trento	30 (11.7%)	8–16.2			42 (10.8%)	7.9–14.3		
Venice	54 (21.0%)	16.2–26.5	5		131 (33.7%)	29–38.6	2	
Vicenza	75 (29.1%)	23.7–35.2	1		53 (13.6%)	10.4–17.4		
Verona	1 (0.4%)	0–2.1			22 (5.7%)	3.6–8.4		
**Clinical Symptoms**								
Asymptomatic	226 (87.9%)	83.3–91.7	9		328 (84.3%)	80.3–87.8	2	
Respiratory	2 (0.8%)	0.1–2.8			10 (2.6%)	1.2–4.7		
Gastrointestinal	6 (2.3%)	0.9–5			1 (0.3%)	0–1.4	1	
Ectoparasites	8 (3.1%)	1.4–6		0.93	32 (8.2%)	5.7–11.4		3.29
Cutaneous lesions	12 (4.7%)	2.4–8			9 (2.3%)	1.1–4.3		
Others or multiple	3 (1.2%)	0.2–3.4			8 (2.1%)	1.9–4		

N = number of animals; n.a. = not available; MN: male neutered; FN: female neutered. *p* = *p*-value of the Pearson’s Χ^2^ test. *p* ≤ 0.05 was considered statistically significant.

**Table 2:** Epidemiological and anamnestic details of seropositive dogs. Results SARS-CoV-2 ELISA-test (S/P% values).

Date of Sampling	Sex	Age (years)	Breed	Province	Symptoms	Value
24/08/2021	M	1	Crossbreed	Padua	None	S/P% = 112%
25/08/2021	F	1	Crossbreed	Venice	None	S/P% = 119%
01/09/2021	M	12	Crossbreed	Padua	None	S/P% = 120%
05/10/2021	M	1	Crossbreed	Padua	None	S/P% = 356%
27/10/2021	M	2	Pit Bull	Venice	None	S/P% = 121%
20/01/2022	M	7	Crossbreed	Venice	None	S/P% = 53%*
07/03/2022	F	4	Hound	Vicenza	None	S/P% = 148%
16/05/2022	M	1	Crossbreed	Venice	None	S/P% = 204%
26/07/2022	F	1	Crossbreed	Venice	None	S/P% = 101%

*: Doubtful sample: borderline value according to the reported cut-off. ELISA test cut-off for positivity (S/P% > 50%).

**Table 3:** Description of the positive cats: anamnestic and epidemiological data, molecular, and serological analysis’ results.

Date of Sampling	Sex	Age (years)	Province	Symptoms	Ct OP	Ct R	Serology
20/10/2021	F	1	Venice	None	Ct 34.85 gE Ct 38.33 gN Neg RdRp	n.d	Neg
27/10/2021	M	1	Venice	None	Ct 25.88 gE Ct 32.67 gN Ct 36.44 RdRp	n.d	S/P% = 306%
21/04/2022	M	2.5	Rovigo	None	n.d	n.d	S/P% = 349%
11/05/2022	M	8.5	Venice	GI + EP *	n.d	n.d	S/P% = 124%

OP: oropharyngeal swab, R: rectal swab, gE: envelope protein gene, gN: nucleoprotein gene, RdRp: RNA-dependent RNA polymerase gene, Neg: negative; n.d= not detected; *: Gastrointestinal symptoms (GI) and presence of Ectoparasites (EP).

**Table 4:** Summary of the SARS-CoV-2 positive cat: timeline and laboratory results.

Time of Sampling	Ct OP	Ct R	Serology
**T0**	Ct 34.32 gE Ct 38.99 gN Neg RdRp	Ct 36.68 gE Ct 32.31 gN Neg RdRp	NP
**T1**	n.d	n.d	n.d
**T2**	NP	NP	n.d

OP: oropharyngeal swab, R: rectal swab, gE: envelope protein gene, gN: nucleoprotein gene, RdRp: RNA-dependent RNA polymerase gene, NP = not performed; n.d = not detected.

## Data Availability

Current Research IZSVe 12/19 data are available from the authors of this manuscript following a reasoned request. Data concerning the sequencing of the SARS-CoV-2 genome are deposited at the public archive of the international data bank GISAID (https://gisaid.org/ (accessed on 25 November 2022)).
